# Application of dosimetry tools for the assessment of e-cigarette aerosol and cigarette smoke generated on two different in vitro exposure systems

**DOI:** 10.1186/s13065-016-0221-9

**Published:** 2016-12-09

**Authors:** Jason Adamson, David Thorne, Benjamin Zainuddin, Andrew Baxter, John McAughey, Marianna Gaça

**Affiliations:** British American Tobacco, R&D, Southampton, SO15 8TL UK

**Keywords:** e-cigarette, Microbalance, Nicotine, Borgwaldt, Vitrocell

## Abstract

The diluted aerosols from a cigarette (3R4F) and an e-cigarette (Vype ePen) were compared in two commercially available in vitro exposure systems: the Borgwaldt RM20S and Vitrocell VC10. Dosimetry was assessed by measuring deposited aerosol mass in the exposure chambers via quartz crystal microbalances, followed by quantification of deposited nicotine on their surface. The two exposure systems were shown to generate the same aerosols (pre-dilution) within analytically quantified nicotine concentration levels (p = 0.105). The dosimetry methods employed enabled assessment of the diluted aerosol at the exposure interface. At a common dilution, the per puff e-cigarette aerosol deposited mass was greater than cigarette smoke. At four dilutions, the RM20S produced deposited mass ranging 0.1–0.5 µg/cm^2^/puff for cigarette and 0.1–0.9 µg/cm^2^/puff for e-cigarette; the VC10 ranged 0.4–2.1 µg/cm^2^/puff for cigarette and 0.3–3.3 µg/cm^2^/puff for e-cigarette. In contrast nicotine delivery was much greater from the cigarette than from the e-cigarette at a common dilution, but consistent with the differing nicotine percentages in the respective aerosols. On the RM20S, nicotine ranged 2.5–16.8 ng/cm^2^/puff for the cigarette and 1.2–5.6 ng/cm^2^/puff for the e-cigarette. On the VC10, nicotine concentration ranged 10.0–93.9 ng/cm^2^/puff for the cigarette and 4.0–12.3 ng/cm^2^/puff for the e-cigarette. The deposited aerosol from a conventional cigarette and an e-cigarette in vitro are compositionally different; this emphasises the importance of understanding and characterising different product aerosols using dosimetry tools. This will enable easier extrapolation and comparison of pre-clinical data and consumer use studies, to help further explore the reduced risk potential of next generation nicotine products.

**Graphical abstract:**
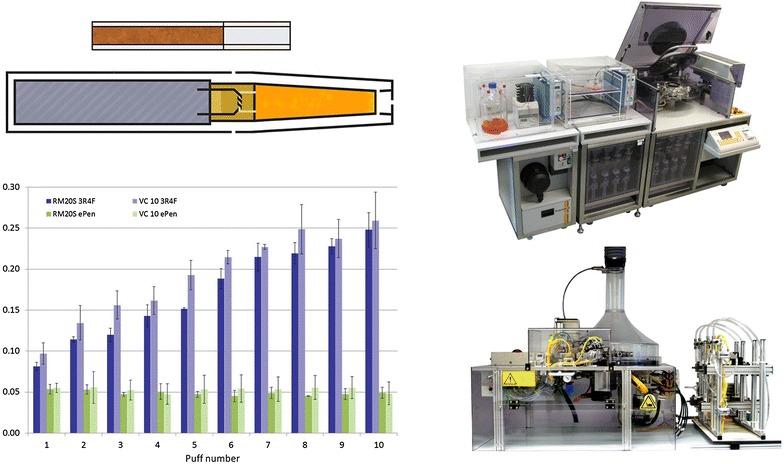
A cigarette and an e-cigarette (top left) were assessed on two different in vitro exposure systems, the Borgwaldt RM20S (*top right*) and the VC 10 (*bottom right*). Compositionally the product aerosols were different, but there was no difference between the same product on different machines (*bottom left*).

A cigarette and an e-cigarette (top left) were assessed on two different in vitro exposure systems, the Borgwaldt RM20S (*top right*) and the VC 10 (*bottom right*). Compositionally the product aerosols were different, but there was no difference between the same product on different machines (*bottom left*).

## Background

In the past decade the awareness and usage of electronic cigarettes (e-cigarettes) has increased exponentially, with over 2.6 million adults using the devices in the United Kingdom as surveyed in 2015 [6]. A study funded by Cancer Research UK further suggests there is now ‘near universal awareness of e-cigarettes’ [9]. Around 12% of Europeans have tried e-cigarettes at some point, and roughly 2% report continued use [13]. The use of electronic-cigarettes and other vapourising devices by those in the United States is also on the rise, with estimations from a recent survey suggesting that 2.6–10% of adults in the US now vape [35]. Public Health England recently reported that compared to cigarettes, electronic cigarettes may be about 95% less harmful and could be a potential aid for smokers trying to quit [27].

The US Food and Drug Administration (FDA) published a draft guidance indicating the scientific studies required to demonstrate significantly reduced harm and risk of nicotine and tobacco products, including the use of in vitro assessment tools [15]. An in vitro aerosol exposure system supports such an approach, where a machine system will generate, dilute and deliver aerosols from cigarettes or e-cigarettes (or other nicotine delivery devices) to cell cultures at the air–liquid interface (ALI) in a chamber or a module, mimicking a physiological aerosol exposure. There are many examples where in vitro tests have been used to assess the biological impact of smoke from tobacco products [7, 8, 22, 23, 25, 29, 31, 32, 40, 41]. But despite the apparent ubiquity of e-cigarettes, in vitro testing has only recently been adopted, and with some equivocal results [10, 28, 30, 36, 37, 42].

The in vitro aerosol exposure environment was established to enable the testing of tobacco smoke and other aerosol products in a more physiologically relevant manner—with whole smoke and whole aerosols delivered to in vitro cultures at the ALI. There are various exposure systems available for such tests, many summarised in Thorne and Adamson [40]. However, most of these commercially available systems were originally designed and intended for use with cigarettes only, well before e-cigarettes and other next generation nicotine and tobacco products became commonplace. These systems can easily be adapted to enable the assessment of e-cigarettes, tobacco heating products (THPs) or even medicinal nicotine inhalers; however careful characterisation of the generated aerosol is required (at the point of generation and at the point of exposure) to enable comparisons before conclusions can be made from the associated biological responses.

There are many and various exposure systems available for the assessment of inhalable products; they differ in size, cost, mechanics, and paired exposure chamber. A complete exposure system requires an aerosol generator, a dilution route and exposure chamber (also called *module*, *plate* or *exposure device* in certain set-ups) in which the biological culture is housed. Some are commercially available and others are bespoke laboratory set-ups [40]. There are certain technical and experimental challenges using next generation nicotine and tobacco products on these traditional smoking machines. These include differences in puffing regimes, greater aerosol density/viscosity, issues with condensation in transit and manual device activation, to name just a few. It is also notable that, although the overall conditions of an exposure system can be controlled in terms of smoke dilution and smoking regimen, it is difficult to measure the actual deposition of smoke on culture inserts [25]. Furthermore, we should not assume that what is known about tobacco smoke aerosol generation, dilution and delivery in such exposure systems will apply to the aerosol of these new products, as their aerosols are not compositionally or chemically the same; exposure must be characterised [39]. Cigarette smoke aerosol has a visible minority particle fraction (5%) suspended within an invisible majority gas and vapour phase in air; this vapour phase comprising principally products of combustion [21]. Looking at next generation nicotine and tobacco products, recent data suggest THP aerosol has a lower vapour phase mass because the tobacco is at sub-combustion temperatures usually <350 °C [38]. E-cigarette aerosol is generated with coil heater temperatures reported as ranging 40–180 °C [11] and is best described as a mist [5]. It is predominately homogeneous particles in air with very low levels of volatile species; in addition to its simpler composition, the e-cigarette aerosol contains substantially lower levels (88 to >99%) of regulatory interest toxicants as compared with tobacco cigarette smoke [26]. Thus quantification of what the cell cultures are exposed to at the interface (the dosimetry) is pivotal in supporting the biological testing of next generation nicotine and tobacco products with such different aerosols.

Dosimetry tools and methods can assess many aspects of the test article’s aerosol and provide important data to relate biological response following exposure to the actual dose of aerosol encountered by the cells (thus confirm aerosol delivery in biological assay systems showing partial or no biological response to exposure). An example would be the direct mass measurement of total deposited particles at the exposure interface, using a quartz crystal microbalance (QCM) device [4]. As particles deposit on the crystal’s surface its mass loading, and thus its natural oscillation frequency, changes which can be converted to an increase in deposited mass. QCMs provide real-time data, are simple to use and are useful for quality assurance purposes too, confirming within an exposure that the culture in the exposure chamber is indeed receiving the aerosol dilution that is being reported. Another example of a dosimetry method complementing QCMs is the quantification of a chemical marker within the surface deposit (of a QCM or a cell culture insert) identifying how much of a certain chemical/compound is being exposed to cells in culture. Nicotine is a good example as it is common amongst the inhalable products we wish to assess. Additionally, there are methods published and in ongoing development to assess components of the vapour phase, such as carbonyl quantification [19, 25] and time of flight mass spectrometry (TOF–MS) [34], as well as trace metal quantification in aerosol emissions [24]. With tools and approaches like these, dosimetry can allow different test products to be directly compared, be employed as a quality assurance tool during exposure and demonstrate physiologically relevant exposure.

**Fig. 1:**
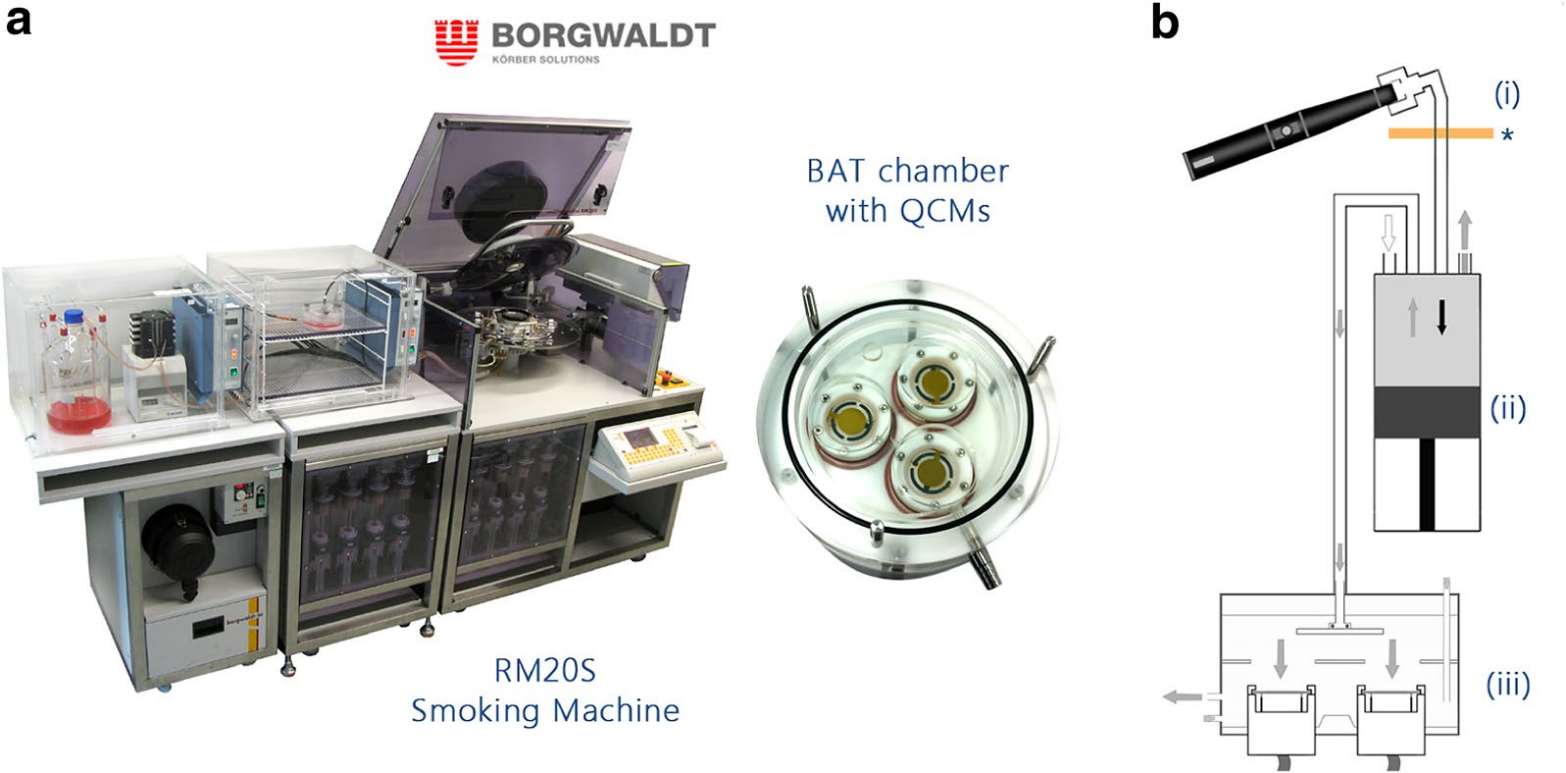
**a** The 8-syringe Borgwaldt RM20S with the BAT exposure chamber (base) installed with three quartz crystal microbalances (QCMs). **b** Cross section of the RM20S; an e-cigarette is shown but the cigarette was puffed in the same way after being lit (*i*). Aerosol was drawn into the syringe where serial dilutions were made with air (*ii*) before being delivered to the exposure chamber (*iii*) where it deposited on the QCM surface. The *asterisked rectangle* under position (*i*) indicates a Cambridge filter pad (CFP)

**Fig. 2:**
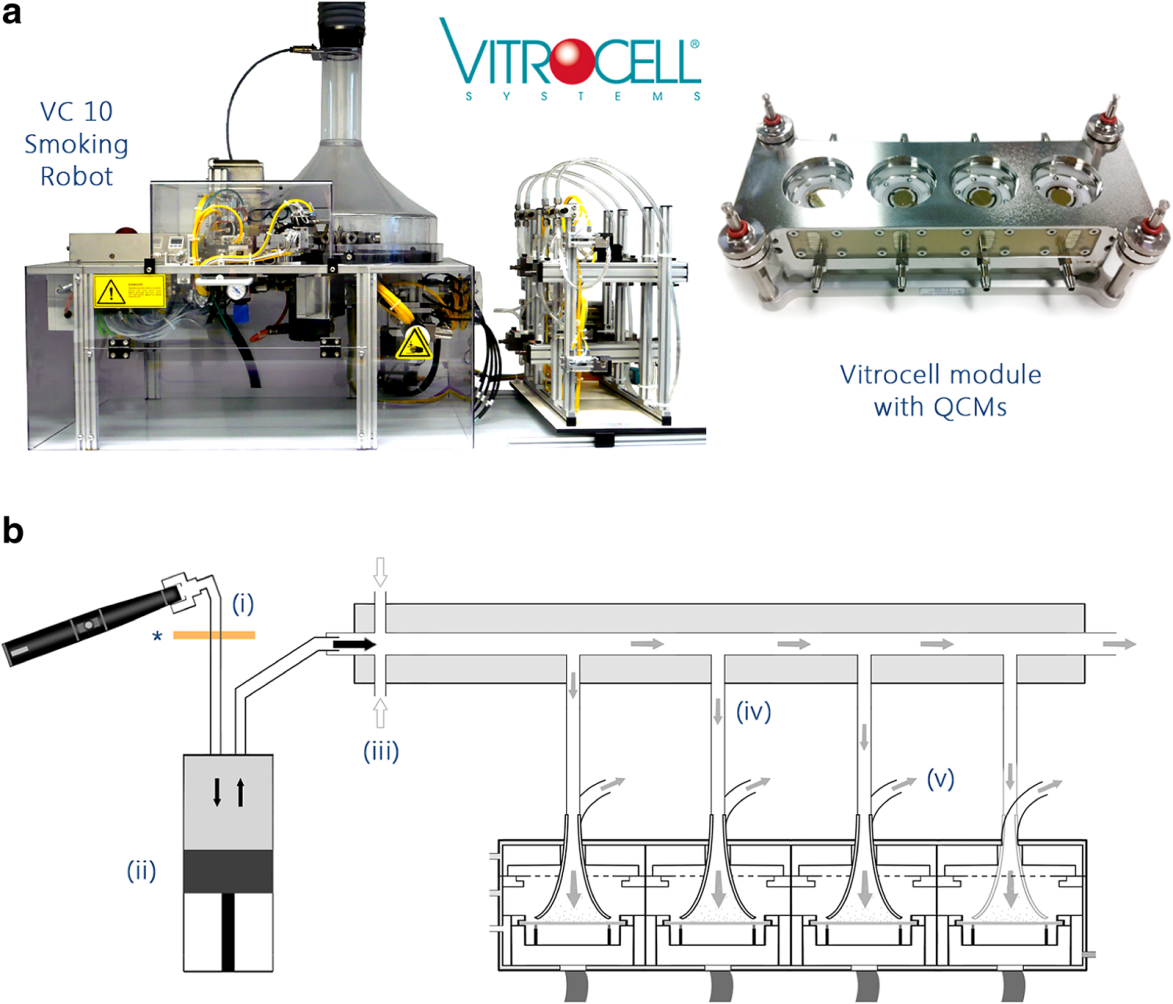
**a** The Vitrocell VC 10 Smoking Robot and 6/4 CF Stainless mammalian exposure module installed with four quartz crystal microbalances (QCMs). **b** Cross section of the VC 10; an e-cigarette is shown here but the cigarette was puffed in the same way after being lit (*i*). Aerosol was drawn into the syringe (*ii*) and delivered to the dilution bar where diluting air was added (*iii*). Diluted aerosol was drawn into the module (*iv*) and deposited on the QCM via negative pressure (*v*). The *asterisked rectangle* under position (*i*) indicates a CFP

The ultimate aim of this study was to compare smoking machine exposure systems and products. Herein we look at two commercially available aerosol exposure systems, the Borgwaldt RM20S (Fig. 1) and the Vitrocell VC 10 (Fig. 2; Table 1). The machines are similar in that they both have a rotary smoking carousel designed to hold and light cigarettes, puff, dilute smoke and deliver it to an exposure chamber housing in vitro cultures. Thereafter they differ in mechanical set-up and dilution principles; the RM20S having 8 independent syringes to dilute aerosol (Fig. 1); the VC 10 having only one syringe which delivers the aliquot of smoke to an independent dilution bar where air is added and a subsample drawn into the exposure chamber via negative pressure (Fig. 2). Both systems are paired with different exposure chambers and these are detailed in Table 2. In overview we can conclude that the systems are largely dissimilar, but achieve the same outcome. Furthermore without dose alignment even the raw data (based on each machine’s dilution principle) are not directly comparable.

**Table 1:** Technical specifications and comparison between the in vitro exposure systems used in this study: Borgwaldt RM20 and Vitrocell VC 10 [40]

	Borgwaldt RM20S smoking machine	Vitrocell VC 10 smoking robot
Dimensions (L × D × H)	2.4 m × 0.8 m × 1.3 m	1.5 m × 0.8 m × 0.85 m
Footprint	Floor standing (2 m^2^)	Bench top (1.2 m^2^)
Dilution system	Syringe based independent dilution system capable of 8 independent dilutions per exposure device	Continuous flow dilution bar capable of 4 independent dilutions per exposure device
Dilution range	1:2–1:4000 (aerosol:air, v/v)	Diluting airflow 0–12 l/min and exposure module vacuum sample rate 5–200 ml/min
Exposure throughput	Up to 8 chambers with 3, 6, 8 inserts/chamber	Up to 4 modules with 3 or 4 inserts/module
Computer controller	Integrated computer	Requires PC
Smoking regime	ISO, HCI, Massachusetts, bell and square (e-cig) puff profiles	ISO, HCI and bespoke (human) smoking profiles, bell and square (e-cig) puff profiles
Tubing transit length to exposure device	~290 cm	~90 cm
Time taken from puff to exposure	~15–24 s (depending on dilution)	~8 s

**Table 2:** Technical specifications and comparison between the two in vitro exposure chambers used in this study: BAT’s exposure chamber and Vitrocell’s mammalian exposure module [40]

	BAT exposure chamber	Vitrocell 6/4 CF Stainless mammalian exposure module
Approximate dimensions	12 cm Ø × 9 cm H	10 cm × 16 cm × 13 cm (D × W × H)
Approximate weight	0.65 kg	4.5 kg
Material	Transparent Perspex^®^	Polished stainless steel, glass and aluminium
Capacity	3 × 24 mm ø culture inserts 6 × 12 mm ø culture inserts 8 × 6.5 mm ø culture inserts 3 × 30 mm ø Petri dishes 1 × 85 mm ø Petri dish	3 or 4 × 24 mm ø culture inserts 3 or 4 × 12 mm ø culture inserts 3 × 35 mm ø Petri dishes
Integrated dose tool	1–3 QCMs	1–4 QCMs
Aerosol delivery to ALI	Sedimentation, Brownian motion	Sedimentation, Brownian motion
Effective residence time	52 s	79 s

Ø = diameter

We have investigated and assessed both exposure systems for deposited aerosol particle mass and nicotine measurements using a reference cigarette (3R4F, University of Kentucky, USA) and a commercially available e-cigarette (Vype ePen, Nicoventures Trading Ltd., UK). Repeatability of aerosol generation was assessed by quantifying puff-by-puff nicotine concentration at source by trapping aerosol on Cambridge filter pads (CFPs) [Figs. 1b, 2b, asterisked rectangles under position (i)]. CFPs are efficient at trapping nicotine which largely resides in the condensed particulate fraction of these aerosols; CFP efficiency for cigarette smoke is stated as retaining at least 99.9% of all particles (ISO 3308:2012), and for e-cigarette aerosols CFPs have been shown to have a nicotine capture efficiency greater than 98% [5]. Exposure interface dose was assessed in two ways: gravimetric mass of deposited particles with QCMs and quantification of nicotine from the exposed QCM surface. In this way the relationship between deposited mass and nicotine concentration across a range of dilutions on two systems could be realised for both products. Finally, these data would allow us to further understand those exposure systems by enabling comparisons between the two types of product aerosols (in terms of mass and nicotine concentration) and importantly, demonstrate delivery of e-cigarette aerosol to the exposure interface.

## Methods

### Test articles—reference cigarette and commercially available e-cigarette

**Fig. 3:**
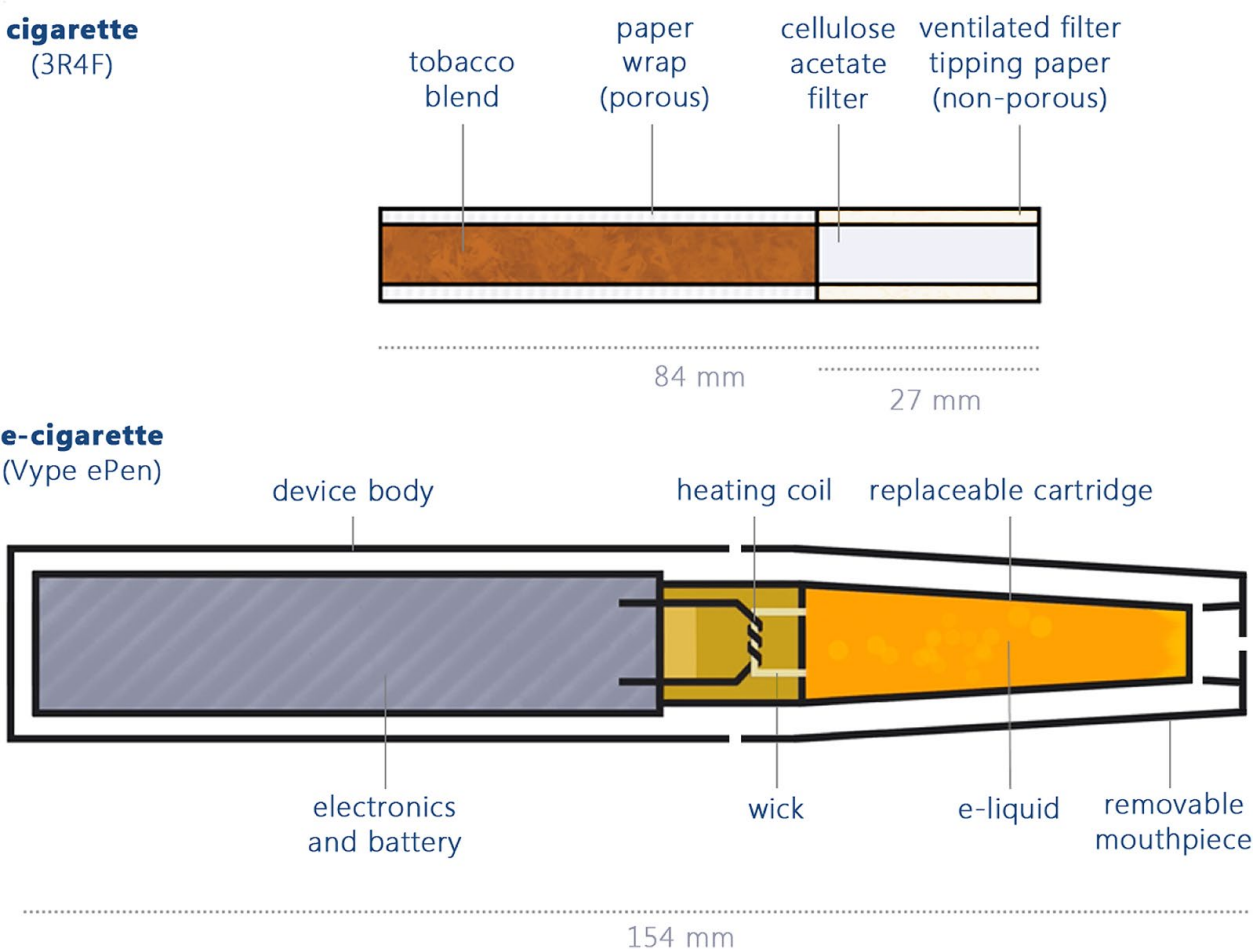
The cigarette and e-cigarette: University of Kentucky reference cigarette 3R4F (0.73 mg pack ISO and 1.97 mg HCI emission nicotine) and e-cigarette (Vype ePen) containing 28 mg nicotine blended tobacco e-liquid (1.58 ml cartridge at 18 mg/ml)

3R4F reference cigarettes (University of Kentucky, USA), 0.73 mg ISO emission nicotine (as stated on the pack) and 1.97 mg measured HCI emission nicotine [12], were conditioned at least 48 h prior to smoking, at 22 ± 1 °C and 60 ± 3% relative humidity, according to International Organisation of Standardisation (ISO) 3402:1999 [18]. Commercially available Vype ePen e-cigarettes (Nicoventures Trading Ltd., UK) with 1.58 ml *Blended Tobacco Flavour* e-liquid cartridges containing 18 mg/ml nicotine were stored at room temperature in the dark prior to use. The basic features of the two test articles are show in Fig. 3.

Per experiment, one cigarette was smoked at the Health Canada Intense (HCI) smoking regime: 2 s 55 ml *bell profile puff* with filter vents blocked, every 30 s [16]. Per experiment, one Vype ePen was vaped (puffed) at the same puffing parameters as the cigarette but with a *square wave profile* instead of bell. The same puffing regime was selected to allow the most appropriate comparison between products and puffs (volume, duration and interval); however the square wave puffing profile is required for e-cigarette vaping to ensure a continuous flow rate for the duration of the puff [17]. With continuous puff flow, aerosol is being generated from the first moment the puff activates; by contrast, if the bell curve profile was employed for e-cigarette puffing, insufficient aerosol would be generated across the puff duration. The e-cigarette (Vype ePen) used in this study is actuated via one of two surface buttons on the device body, high voltage (4.0 V—two arrows pointing towards the mouthpiece) and low voltage (3.6 V—one arrow pointing away from the mouthpiece). High voltage 4.0 V (2.8 Ω, 5.7 W) was used in all experiments, hand-activated 1 s prior to syringe plunging, with a metronome timer used to alert to puffing interval.

### Aerosol generation and exposure: Borgwaldt RM20S smoking machine

For exposure chamber dosimetry, machine smoking/vaping was conducted on the 8-syringe Borgwaldt RM20S, serial number 0508432 (Borgwaldt KC GmbH, Hamburg, Germany) (Fig. 1; Table 1) at four low dilutions of 1:5, 1:10, 1:20, 1:40 (aerosol:air, v:v) as previously described [4]. The study was designed to draw comparisons between systems thus dose selection (low dilutions) was based on maximising deposited particle mass and nicotine concentration in a short duration (10 puffs for all experiments). Each product was smoked/vaped in three independent replicate experiments (n = 3/product). Diluted aerosol was delivered to the exposure chamber housing three quartz crystal microbalances (QCMs) [2]. Aerosol transit length from source to exposure was approximately 290 cm. For collection at source (described fully later), the whole aerosol from each product was trapped by in-line Cambridge filter pads (CFPs) pre-syringe thus no dilution was required.

### Aerosol generation and exposure: Vitrocell VC 10 smoking robot

For exposure chamber dosimetry, machine smoking/puffing was conducted on the Vitrocell VC 10 Smoking Robot, serial number VC 10/141209 (Vitrocell Systems, Waldkirch, Germany) (Fig. 2; Table 1) at four low diluting airflows 0.125, 0.25, 0.5 and 1 l/min, and at an exposure module sample rate of 5 ml/min/well negative pressure as previously described [3]. Airflows were selected based on maximising deposited particle mass and nicotine concentration in a short duration (10 puffs for at source measurements, 5 puffs per product for chamber deposition measurements); furthermore, the airflow range is consistent with other Vitrocell module studies [25]. Each product was smoked/vaped in three independent replicate experiments (n = 3/product). Diluted aerosol was delivered to the exposure module housing four QCMs [3]. Aerosol transit length from source to exposure was approximately 90 cm. For collection at source (described next) the whole aerosol from each product was trapped by in-line CFP pre-syringe thus no dilution was required or set.

### Collection of aerosol at source: puff-by-puff

ISO conditioned 44 mm diameter Cambridge filter pads (CFPs) (Whatman, UK) were sealed one each into a clean holder and installed into the aerosol transit line as close to the point of generation as possible (Figs. 1b, 2b, asterisked rectangles). Between puffs the exposed CFP was removed and placed in a clean flask and stoppered; the in-line pad holder was reinstalled with a fresh unexposed CFP and sealed. Thus we collected emissions to quantify nicotine on a per puff basis, for the duration of 10 puffs from each product on both machines. Each product was smoked/vaped in three independent replicate experiments on both machines (n = 3/product/machine). Quantification of nicotine from the stoppered flasks containing CFPs is described later.

### Measurement of deposited particulate mass

Quartz crystal microbalance (QCM) technology (Vitrocell Systems, Waldkirch, Germany) has already been described for both exposure systems (RM20S [2]; VC 10 [3]). Clean QCMs (5 MHz AT cut quartz crystals held between two Au/Cr polished electrodes; 25 mm diameter, 4.9 cm^2^ surface area, 3.8 cm^2^ *exposed* surface area) were installed in their chamber housing units and stabilised (zero point drift stability) prior to exposure. After the last puff, QCMs were left up to an additional 10 min to reach plateau phase, where recorded mass ceased to increase further, as per previously published dosimetry protocols on both machines [2, 3]. The total mass post-exposure, recorded as micrograms per square centimetre (µg/cm^2^) was divided by the total puff number to present dosimetry on a mean per-puff basis (µg/cm^2^/puff).

### Quantification of nicotine

Nicotine quantification by ultra high performance liquid chromatography triple quad mass spectrometry (UPLC-MS/MS) was based on published methods [20, 33]. All standards, QCM and CFP samples were spiked with d_4_-nicotine at a final concentration of 10 ng/ml as internal standard. Exposed QCM crystals were removed from their housing units without touching the deposited surface, and placed in individual flasks. HPLC-methanol was added to each flask: 3 ml for RM20S samples and 2 ml for VC 10 samples (method differences are discussed later). d_4_-nicotine internal standard was added to each flask (10 µl/ml sample) and shaken for at least 30 min at 160 rpm to wash the surface deposit from the crystal. Thereafter 1 ml of extracts were condensed in an Eppendorf Concentrator 5301 (Eppendorf, UK) for 80 min at 30 °C (higher temperatures degrade the standard). Extracts were resuspended in 1 ml of 5% acetonitrile in water and pipetted into GC vials at 1 ml. The total nicotine quantified on the QCM (ng) was multiplied by the methanol extraction volume, divided by the crystal’s exposed surface area of 3.8 cm^2^ (the *exposed* diameter reduces from 25 mm to 22 mm due to the 0.15 cm housing ‘lip’) and by puff number to present total nicotine per area per puff (ng/cm^2^/puff).

Due to higher predicted source nicotine concentration, exposed CFPs placed in individual stoppered flasks were extracted in 20 ml HPLC-methanol. An additional 200 µl d_4_-nicotine internal standard was added to each flask (10 µl/ml sample consistent with QCM samples) and shaken for at least 30 min at 160 rpm to wash the trapped material from the pad. Thereafter 500 µl of extracts were condensed in an Eppendorf Concentrator 5301 (Eppendorf, UK) for 80 min at 30 °C. Extracts were resuspended in 1 ml of 5% acetonitrile in water and pipetted into GC vials at 500 µl with an additional 500 µl 5% acetonitrile in water. The quantity of nicotine was determined using a Waters Acquity UPLC (Waters, Milford, MA) connected to an AB Sciex 4000 Qtrap MS/MS using Analyst software. An Acquity UPLC HSS C18 column (particle size 1.7 µm, column size 2.1 × 50 mm) was used and the column temperature was maintained at 40 °C. The standards and samples were resolved using a gradient mobile phase consisting of 5 mM ammonium acetate and acetonitrile; the flow rate was 0.5 ml/min. The accuracy was evaluated by comparing the sample peak heights to a calibration curve of known nicotine concentrations ranging from 1 to 1000 ng/ml internal standard for the QCMs, and 10–10,000 ng/ml internal standard for the CFPs. The acceptance criteria for the accuracy of the calibration curve was 100 ± 20%, the LOD was determined from standard deviation values of the signal to noise ratio of the calibration curve greater than 3:1, and the LOQ greater than 10:1.

### Graphics, analysis and statistics

**Fig. 4:**
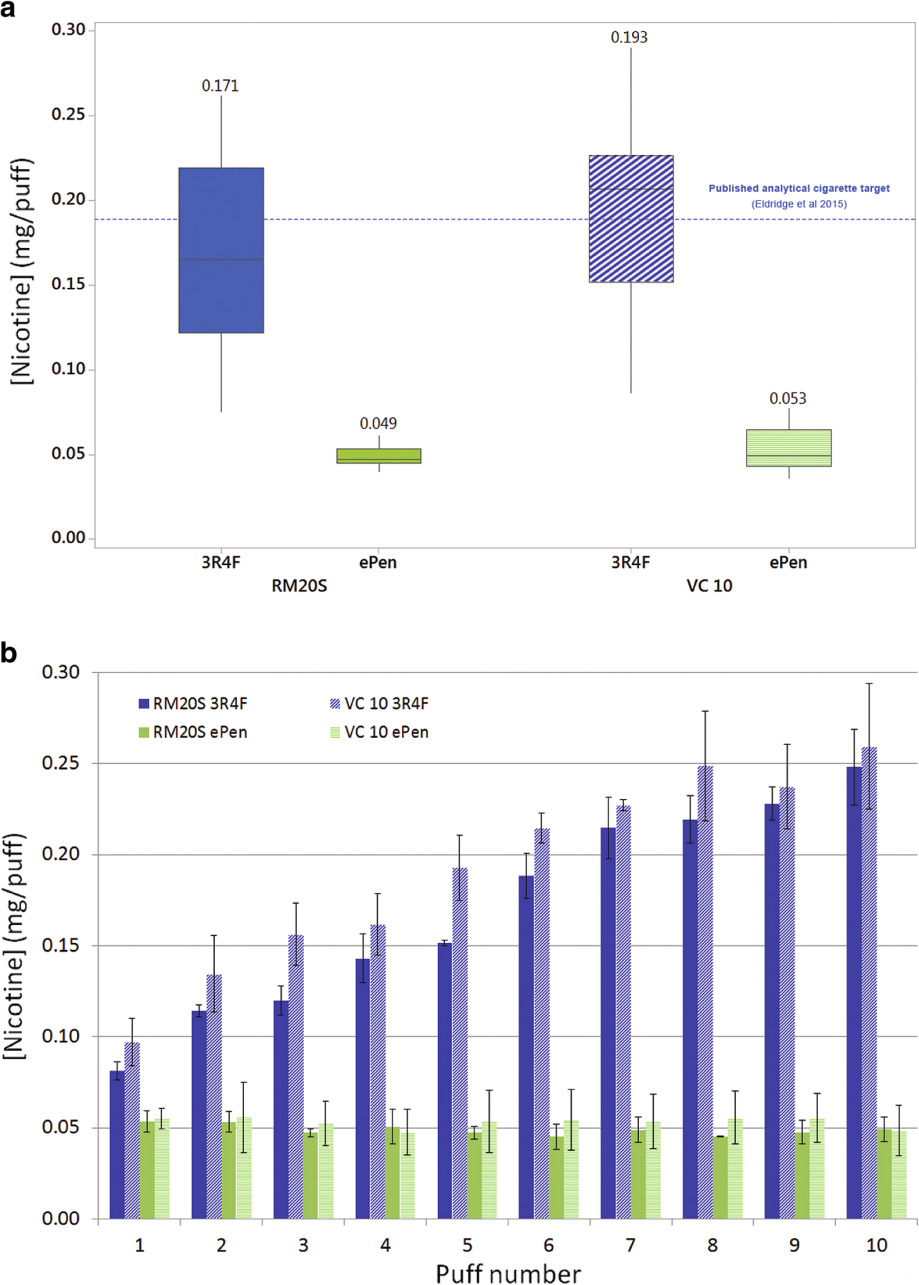
**a** *Boxplot* showing mean nicotine concentration per puff at source from two products on two machines (n = 30/product/machine). The *dotted line* represents the published cigarette mean analytical target value. There was no significant difference between the same products tested on both machines: p = 0.105. The e-cigarette (mean) delivers 3.5 and 3.6 times lower nicotine concentration versus the cigarette (mean) on the RM20S and VC 10 respectively. **b** Individual nicotine values showing the puff-by-puff profile from two products on two machines (n = 3); p ≤ 0.01 for cigarette puffs 1–10 on both machines, p = 0.284 and p = 0.530 for ePen puffs 1–10 on the RM20S and VC 10 respectively

**Fig. 5:**
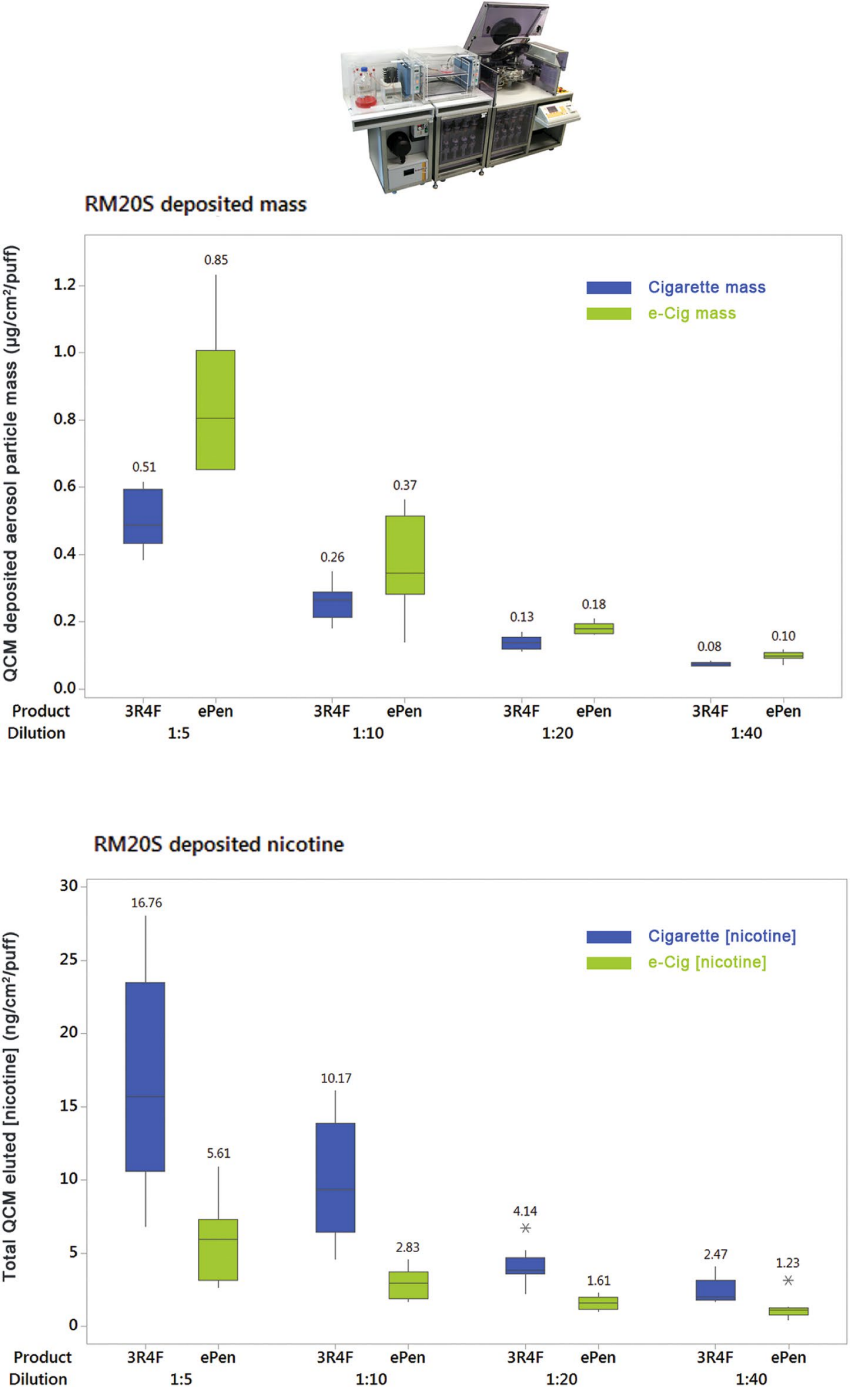
*Boxplot* showing QCM determined aerosol particle deposition from a cigarette and an e-cigarette on the RM20S (*top*). Deposited nicotine concentration from the washed QCM for a cigarette and an e-cigarette on the RM20S (*bottom*). Mass and nicotine values are the mean of three QCMs per chamber and three replicate experiments per product and dilution. *Asterisks* denote single data point outliers, as determined by Minitab

**Fig. 6:**
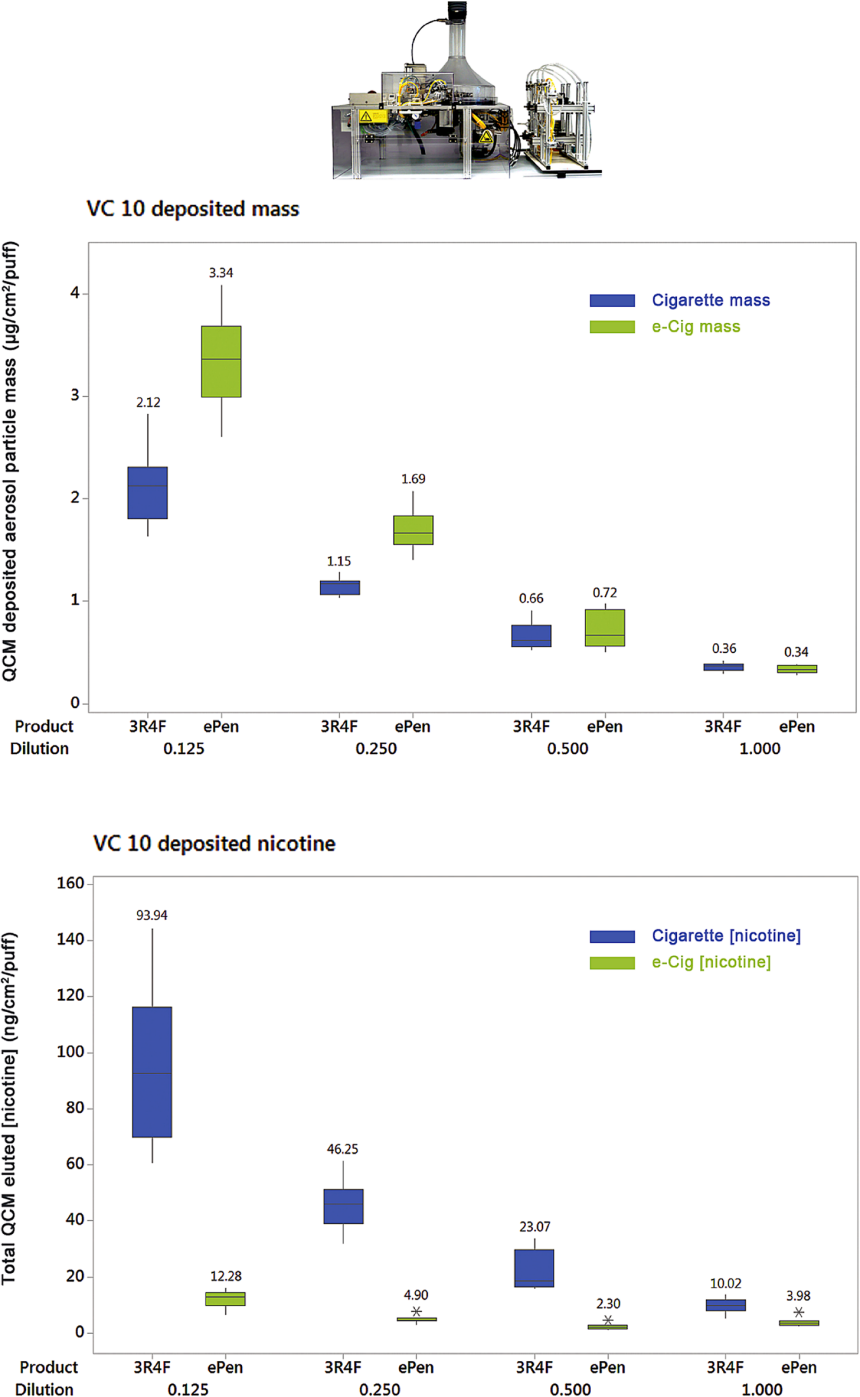
*Boxplot* showing QCM determined aerosol particle deposition from a cigarette and an e-cigarette on the VC 10 (*top*). Deposited nicotine concentration from the washed QCM for a cigarette and an e-cigarette on the VC 10 (*bottom*). Mass and nicotine values are the mean of four QCMs per exposure module and three replicate experiments per product and dilution. *Asterisks* denote single data point outliers, as determined by Minitab

**Fig. 7:**
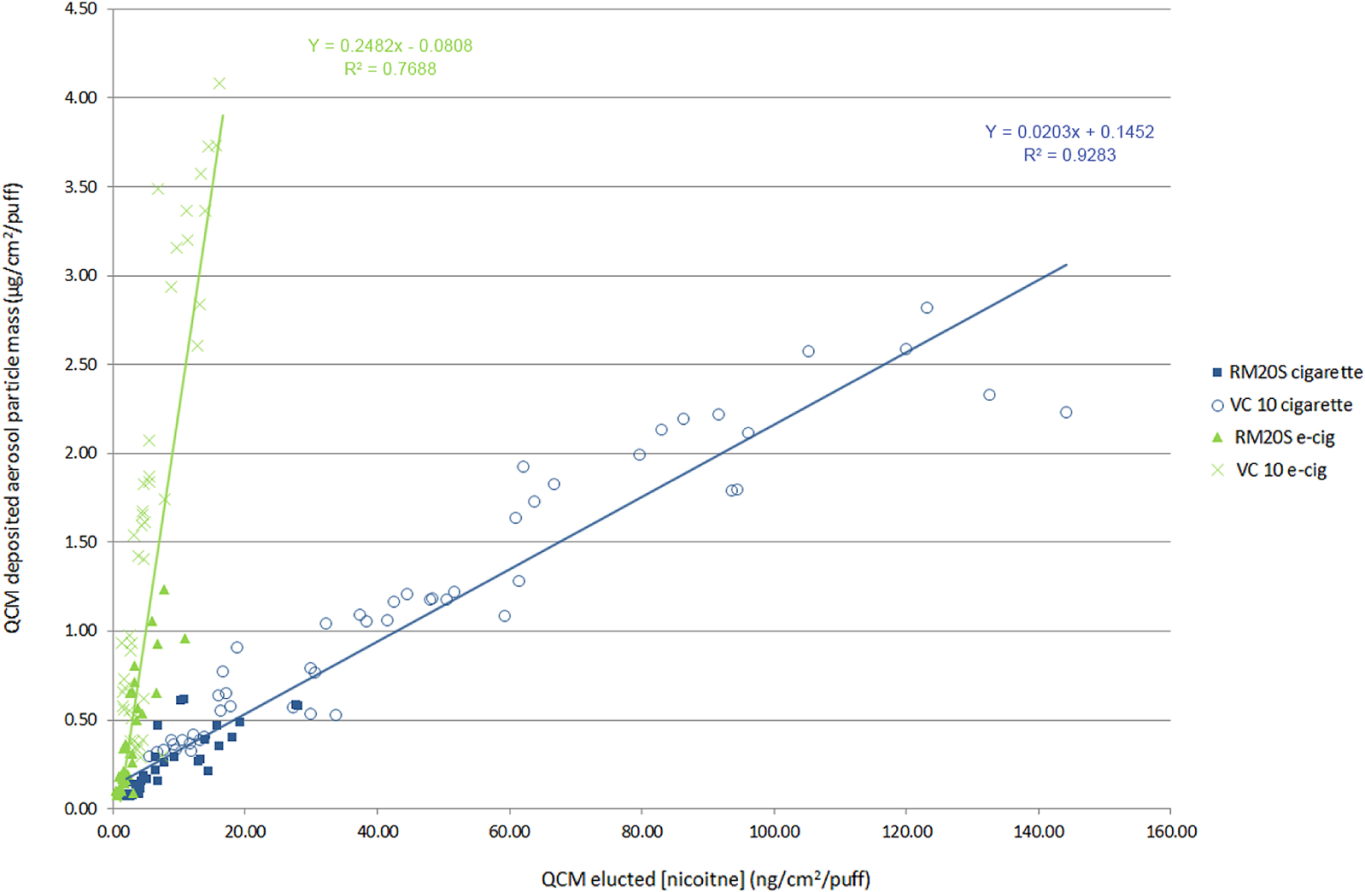
Relationship between deposited mass and nicotine concentration. Data from both exposure systems were combined. Cigarette (*solid squares* RM20S and *open circles* VC 10) R^2^ = 0.928 (Y = 0.0203x + 0.1452); e-cigarette (*solid triangles* RM20S and *crosses* VC 10) R^2^ = 0.769 (Y = 0.2482x − 0.0808)

All raw data and data tables were processed in Microsoft Excel. The boxplots for source nicotine and interval plots for deposited mass and nicotine (Figs. 4a, 5, 6) were produced in Minitab 17. The puff-by-puff source nicotine chart and regression for mass and nicotine (Figs. 4b, 7) were produced in Excel. Comparisons of mean source nicotine from products on different machines were conducted in Minitab by ANOVA test, with the ‘product’ (experimental repeat) as a random effect and nested within ‘machine’; differences between puff numbers for the same product were compared with a General Linear Model, non-nested with ‘product’ as a random effect again. A *p* value <0.05 was considered significant. Irrespective of exposure (total puff number) or nicotine extraction volume, all total deposited mass and nicotine data were normalised to surface area per puff.

## Results

We wanted to attain confidence in aerosol generation repeatability prior to assessment of exposure chamber dosimetry; this was to ensure there were no differences between the two smoking machines for aerosol generation to begin with. Mean nicotine concentration per puff was quantified at source (100% aerosol) by in-line trapping with a CFP (n = 3/puff/product/machine). Mean 3R4F cigarette smoke nicotine concentration was 0.171 ± 0.055 mg/puff on the RM20S and 0.193 ± 0.055 mg/puff on the VC 10. For the e-cigarette, mean nicotine concentration at source was 0.049 ± 0.006 mg/puff on the RM20S and 0.053 ± 0.012 mg/puff on the VC 10 (3.5 and 3.6 times less than the cigarette respectively) (Fig. 4a; Table 3). The mean analytical value for 3R4F reference cigarette nicotine concentration per puff at the HCI regime was published at 0.189 mg/puff (1.97 mg/cig at 10.4 puffs/cig) [12]. As demonstrated, our obtained source nicotine data per puff for the cigarette on both machines was at the expected analytical values previously obtained (Fig. 4a dotted line). For the e-cigarette, in-house measurements have recorded 0.032 mg nicotine per puff for the 55:3:30 regime at low voltage, and 0.0552 mg nicotine per puff for the 80:3:30 regime at high voltage. As we can see here, the puffing parameters (specifically the puff duration and square profile instead of bell) and voltage settings play a significant role in aerosol nicotine delivery. Our e-cigarette aerosols was generated at 55:2:30 high voltage, but our mean nicotine concentrations at source sit reasonably between the two measured values at regimes/voltages above and below. There was no statistically significant difference in nicotine concentration between machines; p = 0.105 (for the two products tested). In generating per puff data we observed the cigarette concentration of nicotine increase from puff 1 to puff 10 as expected; the tobacco rod itself also acts as a filter where tar and nicotine will deposit down the cigarette, enriching the distillable material in the distal rod for later puffs (p ≤ 0.01 for both machines). Yet in contrast and again as predicted, the e-cigarette nicotine concentration per puff was highly consistent in delivery from puff 1–10; p = 0.284 for ePen on the RM20S and p = 0.530 for ePen on the VC 10 (Fig. 4b).

**Table 3:** Cigarette and e-cigarette nicotine concentration per puff at source (pre-dilution) on both machines at the 55:2:30 regime; mean ± standard deviation (n = 30 puffs/product/machine)

	Analytical target (mg/puff)	Borgwaldt RM20S (mg/puff)	Vitrocell VC 10 (mg/puff)
3R4F cigarette	0.189^a^	0.171 ± 0.055	0.193 ± 0.055
ePen e-cigarette	N/A for this regime	0.049 ± 0.006	0.053 ± 0.012

^a^Eldridge et al. [12]

Deposited particle mass was recorded with QCMs at a range of dilutions in the most concentrated range on the Borgwaldt RM20S [1:5–1:40 (aerosol:air, v:v)] and a dose response was observed for both products whereby deposited mass decreased as aerosol dilution increased. For the cigarette, deposited particle mass ranged from 0.08 to 0.51 µg/cm^2^/puff. For the e-cigarette deposited particle mass in the same range was higher at 0.10–0.85 µg/cm^2^/puff [Fig. 5 (top); Table 4]. Those directly exposed quartz crystals were then analysed for nicotine and the same dose–response relationship was observed with dilution. For the cigarette, QCM deposited (quartz crystal eluted) nicotine concentrations ranged 2.47–16.76 ng/cm^2^/puff; for the e-cigarette QCM deposited nicotine concentrations were in the range 1.23–5.61 ng/cm^2^/puff [Fig. 5 (bottom); Table 4]. Deposited particle mass and nicotine concentration was assessed on the Vitrocell VC 10 in the same way, in the range of dilutions 0.125–1.000 l/min (with a 5 ml/min exposure module sample rate by negative pressure). All measured values were higher than the RM20S. A dose response was observed for both products whereby deposited mass decreased as aerosol dilution increased. For the cigarette on the VC 10, deposited particle mass ranged from 0.36 to 2.12 µg/cm^2^/puff. For the e-cigarette, deposited particle mass in the same dilution range was 0.34–3.34 µg/cm^2^/puff [Fig. 6 (top); Table 5]. As before, those directly exposed QCMs were then analysed for nicotine. For the cigarette, QCM deposited (quartz crystal eluted) nicotine concentrations ranged 10.02–93.94 ng/cm^2^/puff; for the e-cigarette QCM deposited nicotine concentrations were in the range 3.98–12.28 ng/cm^2^/puff [Fig. 6 (bottom); Table 5].

**Table 4:** Mean deposited mass (µg/cm^2^/puff) ± standard deviation and mean nicotine concentration (ng/cm^2^/puff) ± standard deviation from the RM20S; three QCMs per chamber and three replicate experiments per product and dilution

Dilution (1:X)	1:5		1:10		1:20		1:40	
Product	3R4F	EPen	3R4F	EPen	3R4F	EPen	3R4F	EPen
Mean mass	0.51 ± 0.09	0.85 ± 0.21	0.26 ± 0.05	0.37 ± 0.14	0.13 ± 0.03	0.18 ± 0.02	0.08 ± 0.01	0.10 ± 0.01
Mean mass ratio	0.60		0.70		0.74		0.81	
Mean (nicotine)	16.76 ± 7.42	5.61 ± 2.78	10.17 ± 4.13	2.83 ± 1.03	4.14 ± 1.25	1.61 ± 0.44	2.47 ± 0.84	1.23 ± 0.77
Mean (nicotine) ratio	2.99		3.60		2.58		2.01	

Ratios are between the cigarette and the e-cigarette at each dilution (3R4F/ePen)

**Table 5:** Mean deposited mass (µg/cm^2^/puff) ± standard deviation and mean nicotine concentration (ng/cm^2^/puff) ± standard deviation from the VC 10; four QCMs per module and three replicate experiments per product and dilution

Dilution (l/min)	0.125		0.250		0.500		1.000	
Product	3R4F	EPen	3R4F	EPen	3R4F	EPen	3R4F	EPen
Mean mass	2.12 ± 0.34	3.34 ± 0.42	1.15 ± 0.08	1.69 ± 0.19	0.66 ± 0.12	0.72 ± 0.17	0.36 ± 0.04	0.34 ± 0.03
Mean mass ratio	0.63		0.68		0.92		1.07	
Mean (nicotine)	93.94 ± 25.62	12.28 ± 2.83	46.25 ± 8.69	4.90 ± 1.13	23.07 ± 7.06	2.30 ± 0.92	10.02 ± 2.56	3.98 ± 1.46
Mean (nicotine) ratio	7.65		9.44		10.03		2.52	

Ratios are between the cigarette and the e-cigarette at each dilution (3R4F/ePen)

Ratio differences between the cigarette and the e-cigarette were calculated for mass and nicotine on both machines, to get an insight into the relationship between the two different nicotine delivery products and how their diluted aerosols behaved across both systems. Absolute values between the two exposure systems were clearly different but the *relationship* between products for deposited mass and nicotine was mostly similar and consistent across dilutions and between machines (Tables 4, 5, ratio values). The ratio difference in deposited mass between cigarette and e-cigarette (3R4F/ePen) on the RM20S at the dilutions tested ranged 0.60–0.81. The ratio difference in deposited nicotine concentration between cigarette and e-cigarette on the RM20S at the dilutions tested was higher and ranged 2.58–3.60. On the VC10, those deposited mass ratios (3R4F/ePen) were in the same range as the RM20S in the lower dilutions (0.125–0.250 l/min) at 0.63 and 0.68 respectively, but diverged from the RM20S in the higher dilutions (0.500–1.000 l/min) at 0.92 and 1.07 respectively. The ratio difference in deposited nicotine concentration between cigarette and e-cigarette on the VC 10 ranged 7.65–10.03 at the first three dilutions but decreased to 2.52 at 1 l/min. These ratio comparisons show agreement at all dilutions on the RM20S; the VC 10 shows parity but there are greater product differences at higher air flow rates and we have previously reported variances in dose delivery from flow rates around 0.5 l/min [1].

A final graphic representation of the linear relationship between deposited mass and nicotine concentration in vitro was produced when all data (from both machines) was plotted for the two products in a regression (Fig. 7). The higher the deposited mass delivered from the cigarette the higher the concentration of nicotine (R^2^ = 0.93); conversely, the e-cigarette delivered a much greater mass and a lower concentration of nicotine in the same dilution ranges tested (R^2^ = 0.77). The chart also confirms the difference in dose delivery between the machines, with the VC 10 (crosses and circles) demonstrating a greater range of mass and nicotine delivery than the RM20S (solid markers), based on the low dilutions chosen for this study (Fig. 7).

## Discussion

As part of a weight of evidence approach, the in vitro exposure of a biological system to inhalable aerosols is one way of generating data to assess the potential of novel nicotine and tobacco products to demonstrate reduced risk. Such products include e-cigarettes: from disposable single-piece cigarette-like products, to modular devices with interchangeable parts, all available in a wide range of e-liquid flavours, ratios of solvent (glycerol:propelyne glycol) and nicotine concentration; and tobacco heating product (THP) devices: in which tobacco can be heated up to (but not usually above) 350 °C releasing nicotine and tobacco flavour with a reduced toxicant profile in the aerosol.

In this study, we aimed to characterise the generation and delivery of a commercially available e-cigarette (Vype ePen) aerosol compared to reference 3R4F cigarette smoke in two in vitro exposure systems: the Borgwaldt RM20S Smoking Machine and the Vitrocell VC 10 Smoking Robot (Figs. 1, 2). Having two different exposure systems with different modes of operation allows us the benefit of a greater understanding of the aerosol exposure environment. Aerosol generation was assessed by trapping with Cambridge filter pads (CFPs) at source and quantification of puff-by-puff nicotine concentration by UPLC-MS/MS. Diluted aerosol deposition at the exposure interface was characterised in the exposure chamber (RM20S) and exposure module (VC 10) by measuring deposited particle mass with QCMs and then quantifying the deposited nicotine concentration per puff from their exposed surfaces by UPLC-MS/MS.

Source nicotine generation per puff for both products were in the region of expected analytical values previously obtained (Table 3; Fig. 4a). This is a positive outcome demonstrating that aerosol generation for in vitro exposure is comparable to that from analytical smoking machines; in addition our nicotine quantification method has been adapted for our purposes and again differs from analytical methods. It was noted that with the cigarette the concentration of nicotine increased per puff, as predicted, yet with the e-cigarette nicotine concentration per puff was largely consistent in delivery. There was no statistically significant difference in mean nicotine concentration between products on different machines, p = 0.105. Mean values were obtained from 10 puffs and as is known there are significant puff-to-puff differences as the tobacco rod shortens, hence larger standard deviation and significant difference between successive puffs 1 through 10, p ≤ 0.01 (Fig. 4b). The e-cigarette displayed high repeatability in the puffing profile, and low puff-to-puff variability resulting in a tighter standard deviation and no significant difference between successive puffs 1 through 10, p = 284 and 0.530 for the RM20S and VC 10 respectively (Fig. 4b). In addition to statistical conclusions, we can also see that the obtained mean value for the cigarette on both machines was in the region of previously reported analytical targets (Fig. 4a) [12].

At the exposure interface (in the exposure chamber) the QCM results show that the e-cigarette delivered higher deposited mass but lower nicotine at a given dilution, whereas the reference cigarette delivered lower mass and much higher concentrations of nicotine at the same dilution as the e-cigarette (Figs. 5, 6 and 7). This is to be expected when we reconsider the compositional and chemical differences between aerosols; it is consistent with the differing nicotine percentages in the respective products. Deposited mass and nicotine show a concentration dependent relationship with both products on both machines. For the cigarette, an R^2^ value of 0.93 was observed; this linear correlation between trapped nicotine and smoke concentration was also observed by Majeed et al. [25], R^2^ = 0.96 (albeit using a different Vitrocell exposure module and set-up). For the e-cigarette, a lower R^2^ of 0.77 gives some doubts over linearity and might suggest there are evaporation effects at very high dilutions. This could be device and/or e-liquid specific and needs further investigation. Assessing different product aerosols within different exposure systems highlights the importance of dosimetric characterisation. These exposure systems were originally designed for use with combustible products in mind. For e-cigarette aerosols, noteworthy differences to cigarette smoke in such systems include visibly wetter aerosols condensing in transit tubing (possibly restricting aerosol flow and impeding syringe function) and some concerns with device button activation synchrony (either manually, or automated with a separate robot) with the syringe puffing to ensure the entire puff is activated and delivered. It is important to be aware of issues such as consistency of device activation and puffing as it will affect dose. A lot of these observations will also change depending on e-cigarette device type/design, e-liquid composition, device battery power and activation voltage, coil resistance, exposure system, transit tubing length and so on. Thus it is crucial to understand each unique set-up and test article prior to in vitro biological exposure. With applied dosimetry, such differences between systems, test articles, cell types and exposure duration become less relevant when biological responses can be presented and aligned against a common dose metric. The differences we observed in delivery between the two exposure systems are likely due to their engineering and dilution mechanisms (Table 1) as we have shown that generation at source *was* consistent between systems for the same product. The VC 10 demonstrated greater values for deposited mass (and thus nicotine concentration) (Fig. 7) and also greater ratio differences between products compared to the RM20S, however their transit lengths from generation to exposure differ too, with the VC 10 being shorter than the RM20S, at 90 and 290 cm respectively. In addition, not only flow rate, but also droplet diameter, diffusion, and gravitational settling play a significant role in the process of aerosol deposition in the Vitrocell^®^ exposure module [25]. Despite these system differences, there was an apparent dose range overlap where 1:5 and 1:10 on the RM20S were approximate to 0.5 and 1.0 L/min on the VC 10, respectively (Figs. 5, 6). These observations can assist when comparing varied biological response data from our two systems. This approach will become even more important when comparing reported data from an ever varied source of test articles, biological endpoints and exposure systems: dosimetry techniques will be able to unite data and systems with diverse modes of dilution.

There are numerous and important chemical markers present in cigarette and e-cigarette aerosol which can be used to characterise dosimetry. In the first instance, nicotine was chosen as an appropriate dosimetric marker: it is a cross-product category chemical which is common between cigarettes, e-cigarettes, THPs, shisha tobacco, oral tobaccos, pipe and loose tobaccos, and medicinal nicotine inhalers. In addition nicotine quantification is reasonably simplistic compared to that of other more complex, trace or volatile chemical compounds such as those found in the vapour phase of tobacco smoke. Data in this study were presented on a ‘per puff’ basis, this being deemed the lowest common denominator for comparison across products which are consumed differently. In vitro a cigarette is usually machine smoked to butt length for around 10 ± 2 puffs/stick (cigarette and smoking regime dependent) whereas a single e-cigarette (Vype ePen in this case) with full e-liquid cartridge can be vaped (puffed) at the same regime as the cigarette in excess of 200 puffs, depending on usage patterns [26]. We also know from behavioural observations and nicotine pharmacokinetic studies that people consume different nicotine delivery products in different ways. A regular combustible cigarette usually delivers a nicotine peak of 18–20 ng/ml in blood plasma shortly after smoking; one early study of e-cigarette use by naive e-cigarette consumers observed much lower peak plasma nicotine values of 1–3 ng/ml [43]. Another study suggested higher nicotine plasma levels up to 23 ng/ml could attained after using e-cigarettes, though taking much longer to peak versus a cigarette [14]. Thus we already start to see a diversity of results and responses within the e-cigarette category. Knowing that people interact with these products differently gives an added justification for normalising in vitro data to ‘per puff’.

There are a few considerations to this study which the authors acknowledge. To compare generation of aerosol at source between the two systems the experimental design was balanced: all products on both machines were puffed 10 times and pads containing the trapped nicotine were washed in 20 ml methanol and spiked with 200 µl d_4_-nicoitne. However, for the comparison of deposited mass and nicotine at the exposure interface (in the chamber) all RM20S data on all product aerosols were generated at 10 puffs and QCMs washed in 3 ml methanol, and for the VC 10 data all product aerosols were generated at 5 puffs and their QCMs washed in 2 ml methanol. This was due to the evolution and improvement of our methods during the duration of this study. The implication for the VC 10 e-cigarette data is minimal, as we demonstrate that delivery from the Vype ePen device is similar for all puffs at source (Fig. 4b). Five minute run times (instead of 10 min) probably had a greater implication on VC 10 cigarette data, as mean puff number was divisible by 5 puffs rather than 10, omitting the latter, higher delivery puffs (Fig. 4b); it could be predicted that mean absolute deposited mass from the cigarette in the VC 10 exposure module be even higher then described here at 5 puffs. However, it is noted that the tar:nicotine ratio for the 3RF4 cigarette is consistent for the two systems (Fig. 7). We observed one anomaly in deposited nicotine from the ePen on the VC 10: delivery was substantially different at the highest dilution, delivering more nicotine at 1 l/min than at 0.5 L/min despite delivering lower mass (Fig. 6). At these two dilutions on the VC 10 we made repeat measurements on numerous occasions and generated the same values for nicotine each time. Because these runs were based on 5 min exposures, the delivery was quite low and therefore prone to overlap between the doses. In our future planned dose work we are repeating nicotine measurements at 1 l/min and will employ an approach for assessment of other next generation nicotine products with longer dose run times of up to 60 min normalised to puff. We predict in this case that the difference between the dilutions may be clearer and in a defined linear relationship. Additionally, anomalies that may be caused by product difference or operator variability will be ironed out by longer duration exposure, where multiple products are consumed per run. These are learnings that will be carried forward into future studies. Another general limitation for us here was the lack of e-cigarette analytical data at the regime we used in this study (55:2:30 high voltage). There are numerous regimes and voltage setting an electronic device can be puffed at, and we have already talked about how puff duration is more important than volume, and that how higher voltage activation results in greater aerosol delivery. Our e-cigarette regime (55:2:30) was selected to make better comparisons with the HCI cigarette regime. Indeed analytical chemistry data at matched regimes will help align in vitro dose data; that said we have shown herein that our exposure systems can produce repeatable aerosol delivery from the Vype ePen under the conditions we selected (Fig. 4). A final note on recording deposited mass data with QCMs: in this study as with our previous dose determination studies [2, 3] we allowed a plateau phase post-exposure for all remaining aerosol in the chamber to deposit; this final value is taken when mass no longer increases and remains stable. We employ this approach to compare varied and new products and exposure systems. During in vitro biological exposure the chamber may be removed from the system directly after the last puff rather than waiting to plateau, and in this instance the remaining aerosol in the chamber will not impact upon the cells. This could result in significantly lower recorded dose values, and anecdotal observations on the RM20S have shown that between run-end and plateau phase the deposited mass value can be up to 2.5 fold greater (data not shown). Again this is not so much of an issue as long as each dose determination method or approach is clearly detailed when presenting the paired biological data. These are all considerations for comparing products, systems and biological endpoints equally and fairly in future investigations.

With the exponential rise of e-cigarette usage [9, 27], the inevitable and rapid evolution of next generation nicotine and tobacco products and our requirement to assess their potential to reduce biological effects in vitro, dosimetry science and applications become more pivotal. Understanding the dosimetry of a given exposure system and the characteristics of the test article aerosol will ensure a better understanding of and confidence in aerosol delivery and biological exposure. We should not assume that the products of the future and their new aerosols will behave the same in these systems as the products before them; it is likely there may be some differences. As for product comparisons, dose to the biological system can be matched by deposited particle mass and/or nicotine concentration (in the first instance). Matching for nicotine concentration will mean that the cell culture is exposed to a greater amount of aerosol from the e-cigarette, pushing the biological system even harder for a response to e-cigarette aerosol comparable to cigarette smoke.

We see the value in dosimetry for all future studies where products will be tested and compared, with dose tools and methods having many applications. We believe these applications could be ranked as follows: first, prove exposure in every experiment (quality assurance) and demonstrate physiologically relevant exposure; then compare and align diverse exposure systems; compare test articles; and finally compare cell types and align biological response data from varied sources. The results reported herein clearly demonstrate that the aerosols generated from both products are not the same, and this makes testing them in vitro challenging, but also interesting and insightful. Indeed both product aerosols look the same, are physically similar and deliver nicotine to the consumer via inhalation, and both have been demonstrated to deliver test aerosol and nicotine in vitro, but how these aerosols are composed and deposit in these exposure systems when diluted with air have been shown to vary. This study emphasises the importance of dosimetry, in understanding the products being tested and the systems they are being tested in. This will facilitate accurate interpretations of biological response data and enable easier extrapolation and comparison of pre-clinical data and consumer use studies.

## Conclusions

The results of our in vitro dosimetry study show that:

- e-cigarette aerosol *is* delivered to and detected at the exposure interface
- at a common dilution, e-cigarette (Vype ePen) aerosol deposited mass is greater than cigarette smoke (3R4F)
- at a common dilution, e-cigarette (Vype ePen) aerosol deposited nicotine concentration is less than cigarette smoke (3R4F) (consistent with emissions)
- deposited mass and nicotine concentration decreases with increased dilution
- irrespective of exposure system, the delivered mass/nicotine relationship is similar for each product; there is no difference between machines (p = 0.105)
- Data from this study help to bridge two dissimilar exposure systems for future products assessment
- despite system differences, there is dose range parity where 1:5 and 1:10 on the RM20S are approximate to 0.5 and 1.0 l/min on the VC 10, respectively
- for the first time we have demonstrated puff-by-puff nicotine concentration generated at source from two in vitro exposure systems, consistent with reported analytical values
- for the first time we have demonstrated a technique to quantify nicotine on the deposited QCM surface, enhancing gravimetric dose

## Abbreviations

ALI: air liquid interface
CFP: Cambridge filter pad
QCM: quartz crystal microbalance
rpm: revolutions per minute
THP: tobacco heating product
UPLC-MS/MS: ultra high performance liquid chromatography-tandem mass spectrometry
v:v: volume:volume
